# Chromoblastomycosis in Timor-Leste: Highlighting an underdiagnosed condition

**DOI:** 10.1016/j.mmcr.2026.100826

**Published:** 2026-10-03

**Authors:** Terlinda da C. Barros, Amadeu R.X.A. Mendes, Dulce MC. Alberto, Manmeet Kaur, Tessa Oakley, Marlous L. Grijsen

**Affiliations:** a Department of Dermatovenereology, Guido Valadares National Hospital, Dili, Timor-Leste; b Ministry of Health, Dili, Timor-Leste; c PathWest Laboratory, Fiona Stanley Hospital, Perth, Australia; d Global and Tropical Health Division, Menzies School of Health Research, Charles Darwin University, Dili, Timor-Leste; e Oxford University Clinical Research Unit Indonesia, Faculty of Medicine Universitas Indonesia, Jakarta, Indonesia; f Centre for Tropical Medicine and Global Health, Nuffield Department of Medicine, University of Oxford, UK

**Keywords:** Chromoblastomycosis, East Timor, Implantation mycosis, Neglected tropical diseases, Skin NTD, Timor-Leste

## Abstract

Chromoblastomycosis is a chronic implantation mycosis caused by dematiaceous fungi, primarily affecting agricultural workers in impoverished (sub)tropical regions. It often causes disfiguring lesions on the lower limbs, leading to stigma, and reduced quality of life. Treatment is prolonged and challenging. We report the first documented case of chromoblastomycosis in Timor-Leste, highlighting the difficulties of managing this neglected disease in remote areas, including limited diagnostic capacity, restricted treatment options, low health literacy, and poor adherence.

## 1 Introduction

Chromoblastomycosis is a chronic subcutaneous implantation mycosis acquired through traumatic inoculation of dematiaceous fungi, most often *Fonsecaea pedrosoi* and *Cladophialophora carrionii*. It is common in tropical, humid regions, particularly among rural farmers [1]. Chromoblastomycosis is a World Health Organization recognized neglected tropical skin disease (skin-NTD); however, reliable estimates of its global burden are lacking [1]. The condition is characterized by verrucous, nodular, or plaque-like lesions that may evolve into ulcerative or tumorous growths. Chromoblastomycosis progresses slowly over years and, if not treated appropriately, often leads to significant morbidity and disfigurement [1]. Diagnosis relies on the detection of sclerotic bodies (or copper pennies) on direct microscopy or histopathological examination of skin specimens, with cultures aiding species identification [2]. Treatment remains challenging: itraconazole or terbinafine are considered first-line therapies, often combined with surgery, cryo- or thermotherapy [3]. In Timor-Leste, limited awareness of the condition and scarce healthcare resources further complicate management. We describe the first reported case of chromoblastomycosis in Timor-Leste, highlighting diagnostic challenges, therapeutic limitations, and broader implications for disease management shared by many low-resourced settings.

## 2 Case presentation

A 49-year-old male farmer from central Timor-Leste presented to Hospitál Nasionál Guido Valadares (HNGV) in Dili, the capital city, with multiple itchy and painful ulcerated plaques on the right leg and arm, which had progressed over more than 30 years and impacted his daily activities (Fig. 1). The symptoms began in 1990 following a thorn injury to the right lower leg. The lesion evolved from a painless papule into enlarging plaques with satellite lesions. The patient relied mostly on traditional therapies, including the application of herbal pastes, decoction baths, and ashes from burned leaves. The lesions expanded, including the appearance of new nodular lesions on the right arm, with no response to oral and topical antibiotic therapy and antifungal creams. Upon presentation at HNGV in 2023, the differential diagnoses included neurodermatitis, chromoblastomycosis, cutaneous tuberculosis, atypical mycobacterial infection and verrucous carcinoma. Ziehl-Neelsen staining and fungal cultures were negative. Histopathology of a skin biopsy, which was analyzed at the Royal Darwin Hospital Anatomical Pathology department in Australia, revealed sclerotic bodies consistent with chromoblastomycosis (Fig. 1). Oral itraconazole was provided over the past two years, at doses ranging from 100 to 200mg twice daily depending on availability. However, treatment was interrupted on multiple occasions for several months because of local drug shortages. Our patient remains under follow-up with substantial improvement of the lesions. He reported significant psychosocial impact, including activity limitation, anxiety and embarrassment, with a Dermatology Life Quality Index (DLQI) score of 15, indicating a very large effect on quality of life. Some community members attribute his condition to a curse or cultural wrongdoing.

**Fig. 1:**
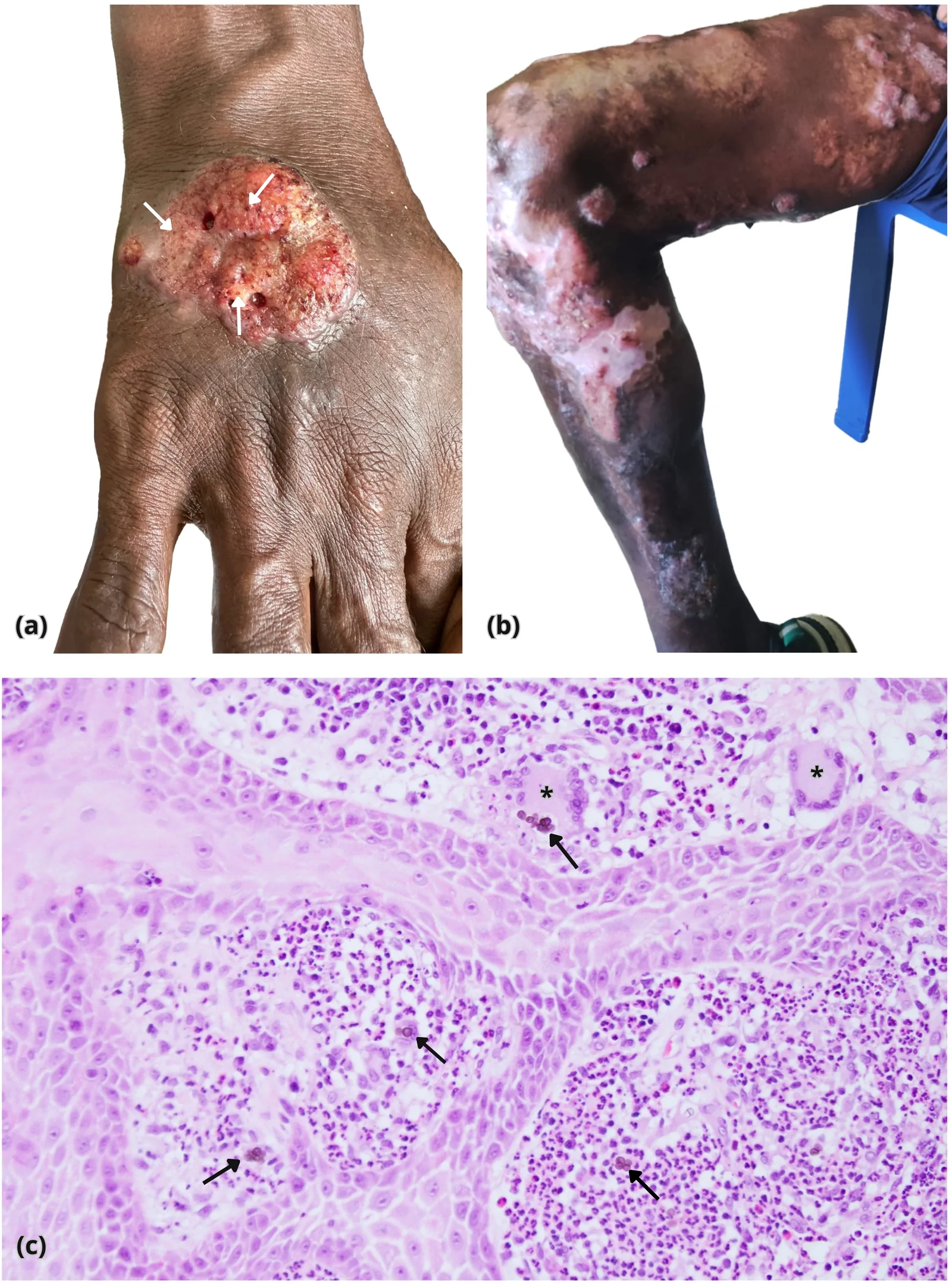
Clinical manifestations and histopathology of chromoblastomycosis in a farmer from Timor-Leste. (a) A verrucous, moist plaque on the right hand with multiple ‘black dots’ (white arrows), a diagnostic clue representing transepidermal elimination of pigmented fungal elements; these sites are ideal for skin scrapings, which will reveal characteristic dark-brown, thick-walled, fungal cells (sclerotic bodies). (b) Verrucous, hyperkeratotic and atrophic tumors and plaques on the right leg. (c) Haematoxylin and eosin-stained section of the dermis showing multinucleated giant cells (*), suppurative neutrophilic microabscesses and sclerotic bodies (black arrows) resembling ‘copper-pennies’ (or Medlar bodies; ×40 magnification).

## 3 Discussion

This case highlights the challenges of recognizing and managing chromoblastomycosis in rural, resource-limited settings. Although endemic in parts of South-East Asia [4], including neighbouring eastern Indonesia [5], this is, to our knowledge, the first reported case of chromoblastomycosis from Timor-Leste. Rather than reflecting true absence, the lack or previous reports more likely reflects limited clinical awareness among healthcare providers, constrained diagnostic capacity and weak surveillance systems. This is further exemplified by the exclusion of fungal NTD, including chromoblastomycosis, from the Skin NTD Strategic Framework for WHO South-East Asia Region 2024-2030, despite their substantial burden in the region [6].

The diagnosis was delayed for many years due to both patient- and system-level barriers. Contributing factors included limited patient health literacy, reliance on traditional medicine, low awareness among healthcare providers, including traditional healers, and insufficient local diagnostic capacity, including microscopy, culture, and histopathology. Definitive confirmation was ultimately performed abroad, adding logistical complexities, further delay and significant financial costs. Direct microscopy using potassium hydroxide (KOH) preparation, for example, could have enabled earlier diagnosis, as the presence of sclerotic bodies is pathognomonic [2].

Treatment recommendations for chromoblastomycosis are often guided by local drug availability and patient tolerability. Therapy is typically prolonged and variable, depending on lesion size, chronicity, adherence, treatment regimen, and access to adjunctive modalities, including surgery, cryotherapy, or immunomodulatory agents like imiquimod, a toll-like receptor 7 agonist that may enhance treatment response [7,8]. Relapse remains common with rates reported as high as 80% [3].

Despite improvement on itraconazole (100–400 mg/day) recurrent treatment interruptions from drug shortages contributed to disease progression and may have increased the risk of developing antifungal resistance, a concern compounded by the unavailability of resistance testing. This case underscores the importance of reliable drug supply chains in low-resource settings, where alternatives like terbinafine and posaconazole, are generally inaccessible.

Chromoblastomycosis imposes substantial socio-economic and psychological burdens, particularly in resource-limited settings. Early case detection is key and can be strengthened by training frontline healthcare workers in common and neglected skin diseases, investing in basic mycological diagnostics, and expanding access to specialist care through teledermatology and digital health platforms. Likewise, reliable essential medicine supply chains, public health education, and regional collaboration for diagnostics and advanced care are essential to improving outcomes. Further surveillance is needed to better understand the disease burden and inform policy, resource allocation, and equitable access to healthcare in under-resourced settings like Timor-Leste, in line with the World Health Assembly's Resolution recognizing skin diseases as a global public health priority.

## CRediT authorship contribution statement

**Terlinda da C. Barros:** Writing – original draft, Data curation, Conceptualization. **Amadeu R.X.A. Mendes:** Writing – original draft. **Dulce MC. Alberto:** Writing – review & editing. **Manmeet Kaur:** Writing – review & editing, Investigation. **Tessa Oakley:** Writing – review & editing, Supervision, Investigation. **Marlous L. Grijsen:** Writing – review & editing, Visualization, Supervision, Conceptualization.

## Consent

The patient provided written informed consent for the publication of anonymized medical information and clinical images.

## Funding

None.

## Conflicts of interest

None declared.
